# Resolving Dilemmas in ICT-Enhanced Interventions: A Cross-Platform-Mediated Strategy

**DOI:** 10.1093/geroni/igab046.723

**Published:** 2021-12-17

**Authors:** Dara K Y Leung, Frankie H C Wong, Gloria H Y Wong, Terry Y S Lum

**Affiliations:** The University of Hong Kong, Hong Kong, Hong Kong

## Abstract

Face-to-face interventions in social care settings are severely disrupted under COVID-19. Previous studies support Information and communication technology (ICT) enhanced intervention as an effective alternative. While difficulties older adults experienced in using ICT were examined extensively, there are fewer discussions on how innate medium characteristics of the delivery mode influence therapeutic interactions. This study explored these embedded challenges in ICT-enhanced psychosocial interventions and possible solutions. We conducted on-site observations and focus groups with 12 participants from two teleconferencing-aided intervention groups for chronic pain with exercise and psychotherapy elements. Observation notes and transcriptions of focus groups recordings were analyzed using thematic analysis. We identified three overarching themes: empowerment, dilemmas, and cross-platform mediated strategy. ICT empowered participants by promoting autonomy and self-management, yet two dilemmas that stemmed from technological affordances undermined the quality of communication. A screen-camera dilemma occurred when participants tried to observe instructions from interventionists while demonstrating their posture in front of the camera for guidance. The blurring boundary between therapy and home settings presented another dilemma. Although teleconferencing increased flexibility and comfort in participation, interruptions from the background environment and intersections of family living spaces disrupted audio-visual communication and jeopardized the sense of security. As a solution, interventionists adopted a cross-platform mediated strategy to bypass the dilemmas. They communicated and delivered supplementary materials through different media, including printed materials and video streamings. Interventionists could consider the unique structural features in different media and the potential impact of participants’ sociodemographic factors, especially those associated with digital literacy.

